# Biomechanics literacy among basketball coaches: implications for long-term athlete development and coaching practice

**DOI:** 10.3389/fspor.2026.1852679

**Published:** 2026-07-03

**Authors:** Hasan Ashkanani, Omar Mahfouth, Hashem A. Kilani, Iyad Yousef, Mahmoud Kayed, Mohammed Mubaideen, Hatem Al-Shlool

**Affiliations:** 1 The Public Authority for Applied Education and Training, Department of Physical Education, Kuwait City, Kuwait; 2 Department of Physical and Health Education, Ahliyya Amman University, Amman, Jordan; 3 Department of Kinesiology, The University of Jordan, Amman, Jordan; 4 Department of Physical Education, Birzeit University, Birzeit, Palestine

**Keywords:** athlete development, basketball coaching, biomechanics knowledge, coach education, LTAD

## Abstract

**Background:**

The Long-Term Athlete Development (LTAD) model emphasizes developmentally appropriate training; however, its effectiveness depends on coaches’ ability to apply scientific knowledge in practice. Biomechanics literacy represents a critical yet underexplored component of coaching competence.

**Purpose:**

This study aimed to assess biomechanics literacy among basketball coaches and examine its potential relevance to LTAD-oriented coaching environments.

**Methods:**

A cross-sectional design was used involving 42 certified basketball coaches in Jordan. The sample consisted of 34 male coaches and 8 female coaches. A 50-item LTAD-based cognitive assessment evaluated knowledge across developmental stages and biomechanical domains. Descriptive statistics, independent samples *t*-tests, and one-way ANOVA were conducted.

**Results:**

Overall biomechanics literacy was low (*M* = 24.52, SD = 9.44; 49.05% of the total score), with all LTAD stages below the predefined 60% threshold. Lower scores were observed in neuromuscular development (44.05%), movement mechanics (47.96%), and injury prevention (47.62%). No statistically significant differences were detected across sex or certification levels (*p* > .05). However, these findings should be interpreted cautiously due to the relatively small sample size and limited number of female participants.

**Conclusion:**

The findings indicate relatively modest levels of biomechanics literacy among basketball coaches across LTAD developmental stages. Strengthening the integration of applied biomechanics education within coach development programs may support evidence-informed coaching practice and LTAD-oriented athlete development.

## Introduction

The integration of scientific knowledge into coaching practice has become increasingly important in optimizing athlete development and performance outcomes (1, 2). Contemporary coaching frameworks emphasize evidence-based practice, where coaches are expected to apply principles from biomechanics, physiology, and motor learning to enhance performance and reduce injury risk (3, 4). Among these disciplines, biomechanics plays a central role in analyzing movement efficiency, technique optimization, reducing injury risk (5, 6), and load distribution during sport-specific tasks (7).

In basketball, performance relies on complex, multi-joint movements that require coordination, force production, and precise timing. Biomechanical principles are therefore essential for improving technical execution and supporting long-term athlete progression (8). The Long-Term Athlete Development (LTAD) model provides a structured framework for guiding athlete progression across developmental stages, emphasizing progressive skill acquisition, training adaptation, and appropriate load management (9, 10). Effective implementation of LTAD requires coaches to integrate biomechanical understanding into training design and performance analysis. The LTAD framework is grounded in the concept of trainability during childhood and adolescence, where specific physical capacities demonstrate heightened responsiveness to training stimuli at particular developmental stages (11).

However, despite the recognized importance of biomechanics, previous research suggests that coaches may demonstrate variability in their understanding and integration of scientific knowledge within coaching environments (12, 13). Limited integration of biomechanics concepts within coach education programs may reduce opportunities for coaches to engage with evidence-informed approaches relevant to movement analysis, training design, and athlete development.

This gap between knowledge and practice may negatively affect coaching effectiveness, particularly in relation to movement correction, injury prevention, and long-term athlete development strategies.

Coach education programs are expected to bridge this gap by developing both theoretical understanding and applied competencies. Nevertheless, recent evidence indicates that coach education often lacks sufficient emphasis on the applied integration of biomechanics into coaching practice (12, 13). This limitation may result in coaches relying on experiential knowledge rather than scientifically grounded approaches, particularly in dynamic team sports such as basketball.

Coaching knowledge and athlete development are also influenced by the broader training environment and organizational culture. Research using the Talent Development Environment Questionnaire (TDEQ) framework highlights the importance of supportive developmental environments in facilitating coach learning and athlete progression (14, 15).

Furthermore, existing research has predominantly focused on general coaching competencies, with limited attention given to domain-specific knowledge such as biomechanics within developmental frameworks (16). As a result, there remains a lack of empirical evidence examining biomechanics knowledge among coaches and its relevance to the implementation of LTAD principles. In the present study, biomechanics literacy refers specifically to coaches' cognitive understanding of biomechanical principles relevant to movement analysis, training design, injury prevention, and athlete development within LTAD-oriented coaching environments. The construct is conceptualized as applied scientific knowledge that informs coaching decision-making rather than direct observation of coaching behavior or practical coaching effectiveness. Accordingly, the present study evaluates coaches' knowledge-based understanding of biomechanics principles and does not directly assess real-world coaching performance or athlete outcomes.

Therefore, the purpose of this study was to assess biomechanics literacy among basketball coaches and examine its potential relevance to LTAD-oriented coaching practice. By identifying knowledge gaps, this study aims to contribute to the improvement of coach education programs and support the integration of scientific principles within coaching environments.

The Long-Term Athlete Development (LTAD) model provides a structured framework for aligning training with athletes’ developmental readiness and promoting sustainable performance pathways (17). Within this framework, biomechanics plays a critical role in optimizing movement efficiency, force production, and neuromuscular coordination across developmental stages.

**Figure 1:**
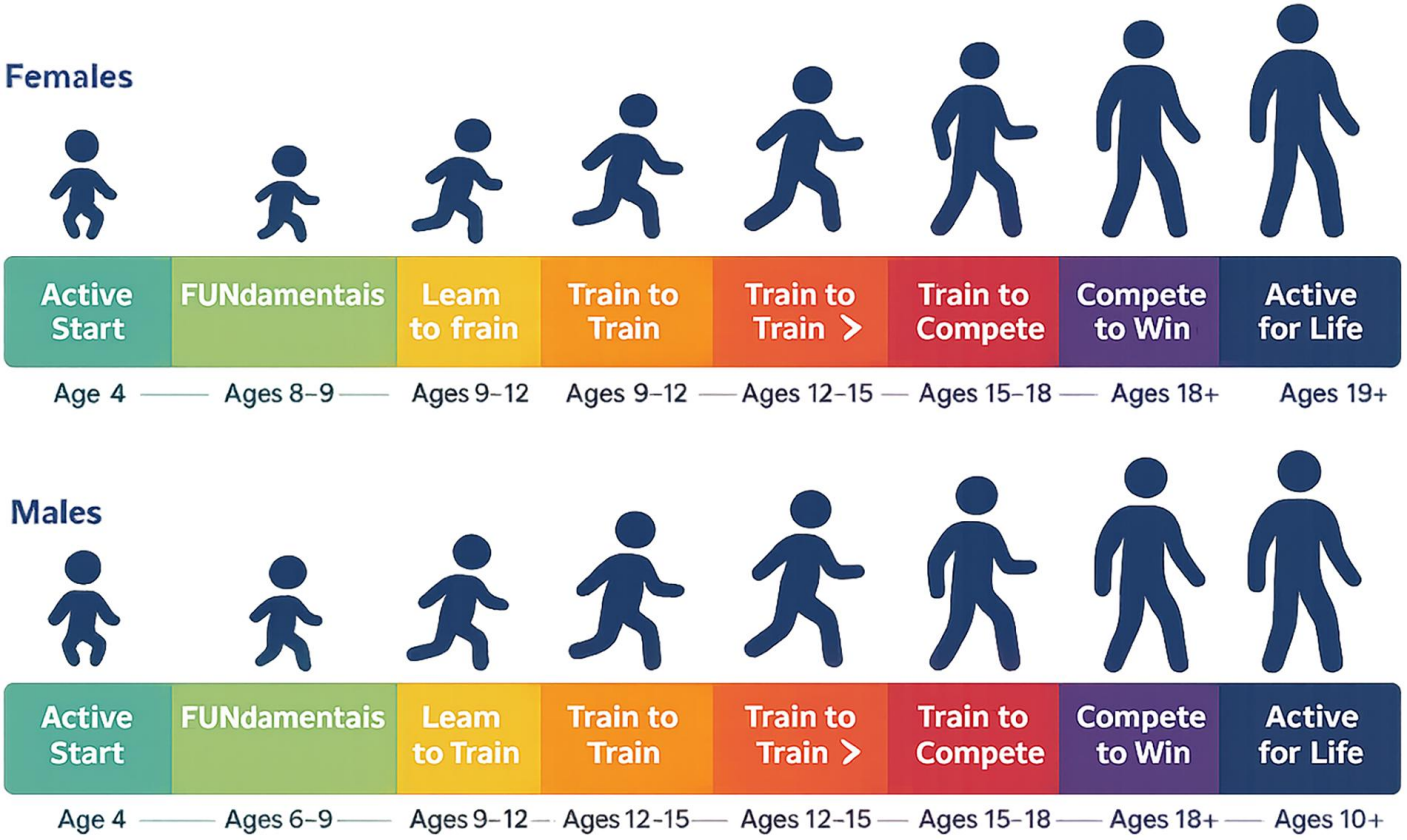
Conceptual representation of the long-term athlete development (LTAD) framework illustrating the progression from physical literacy and foundational movement skills toward sport-specific performance and lifelong athletic participation. Adapted from Balyi et al. (17).

As illustrated in Figure 1, the LTAD model emphasizes a progressive transition from fundamental movement skills and physical literacy in early stages to sport-specific performance and elite training in later stages. This framework emphasizes the alignment between scientific knowledge and coaching practice to support long-term performance development (17, 18). This progression highlights the importance of integrating biomechanical principles throughout athlete development to support both performance enhancement and injury prevention.

## Materials and methods

### Study design

A descriptive cross-sectional survey design was adopted to evaluate biomechanics knowledge within LTAD-oriented coaching environments. This design was considered appropriate given the study's objective of quantifying cognitive understanding across certified coaching populations.

### Study context

The study was conducted in basketball training centers, clubs, and academies across Jordan. Data collection took place between January 8, 2025 and April 10, 2025. Participants were certified basketball coaches registered with the Jordanian Basketball Federation.

### Participants

Participants were certified basketball coaches in Jordan recognized by the Jordanian Basketball Federation and the Youth Leadership Development Center. Coaches were categorized according to the national certification system into three levels:

Level C—beginner coaches
Level B—intermediate coaches
Level A—advanced coaches

These classification thresholds were adopted based on conventional standards in educational assessment and knowledge-based evaluation. The study population comprised 255 officially licensed basketball coaches recognized by the national federation. A purposive sample of 42 coaches (17% of the total population) was recruited from clubs, academies, and training centers across Jordan. The sample consisted of 34 male coaches and 8 female coaches. Because the number of female participants was limited, sex-based comparisons should be interpreted cautiously and were considered exploratory.

Although the sample size was relatively small, it represents a meaningful proportion of the total population of certified basketball coaches in Jordan, supporting the relevance of the findings.

### LTAD-based biomechanics literacy assessment

The instrument was designed to assess applied biomechanics knowledge relevant to coaching practice within LTAD contexts, focusing on coaches' ability to understand and apply scientific principles in sport-specific training environments. Biomechanics literacy was assessed using a structured 50-item LTAD-based cognitive assessment designed to evaluate coaches' understanding of biomechanical principles relevant to coaching decision-making within athlete development contexts. The instrument focused on applied conceptual understanding rather than memorization of isolated biomechanical facts, consistent with pedagogical models emphasizing domain-specific professional knowledge (19).

The instrument evaluated coaches' understanding across the seven LTAD developmental stages and incorporated key biomechanical concepts including force production, kinetic chain integration, balance, coordination, and injury risk management. Items were designed to assess coaches' understanding of movement mechanics, force production, load management, injury prevention, and movement analysis within basketball-specific coaching scenarios. Questions required participants to identify biomechanical principles relevant to practical coaching situations rather than recall isolated theoretical definitions. For example, coaches were asked to identify biomechanical factors associated with landing mechanics, movement efficiency, or force transfer during sport-specific tasks.

An example assessment item included:

“Which biomechanical principle is most important for reducing knee valgus during landing tasks in basketball athletes?”

(A) Base of support
(B) Joint alignment
(C) Angular momentum
(D) Reaction time

Correct responses were determined according to established biomechanical principles and LTAD-related scientific references.

Although the assessment was knowledge-based, item construction emphasized contextualized interpretation of biomechanical concepts within coaching environments rather than simple factual recall.

As shown in Table 1, the test covered key biomechanical domains relevant to coaching practice, linking theoretical knowledge to practical applications in basketball training.

**Table 1:** Biomechanics literacy areas assessed within the LTAD cognitive test.

Domain	Knowledge indicator	Example application in coaching
Movement mechanics	Understanding kinetic chain and joint alignment	Teaching correct squat and landing technique
Force production	Knowledge of power generation and rate of force development	Designing plyometric training
Balance and stability	Awareness of center of mass and base of support	Improving defensive positioning
Coordination	Neuromuscular control during complex movements	Enhancing agility drills
Injury prevention	Safe loading patterns and biomechanical risk factors	Reducing ACL injury risk
Technique optimization	Mechanical efficiency in sport-specific skills	Refining shooting mechanics

### Validity

Content validity was established through expert evaluation. The instrument was reviewed by a panel of nine specialists in sports training and basketball coaching to ensure clarity, relevance, and alignment with LTAD theoretical principles. Based on their feedback, several items were revised before producing the final 50-item version used in the study.

### Basketball Coaches' test

The test included 50 questions distributed across the following LTAD stages:

1. Active Start—7 questions
2. FUNdamentals—8 questions
3. Learn to Train—7 questions
4. Train to Train—10 questions
5. Train to Compete—6 questions
6. Compete to Win—6 questions
7. Active for Life—6 questions

### Biomechanical knowledge dimension

To enhance the analytical depth of the instrument, a biomechanical knowledge dimension was incorporated within the LTAD cognitive test. This dimension evaluated coaches' understanding of fundamental biomechanical principles related to movement efficiency, force production, balance, coordination, injury prevention, and technique optimization. The inclusion of biomechanical knowledge aligns with contemporary coaching frameworks that emphasize evidence-based training practices and the scientific analysis of athletic movement.

### Instrument reliability

Instrument reliability was assessed using a test–retest procedure with a pilot sample of 14 basketball coaches. Item difficulty and discrimination indices were calculated to evaluate test quality. Internal consistency was estimated using the Kuder–Richardson reliability coefficient (KR-20), which yielded a reliability value of 0.763, indicating acceptable internal consistency. (Table 2) This value indicates acceptable internal consistency for knowledge-based assessment tools in sport science research.

**Table 2:** Reliability analysis, difficulty indices, and discrimination indices derived from the pilot sample (*N* = 14).

Stage	No. of questions	*M*	SD	Reliability
Active start	7	3.16	0.66	.824
FUNdamentals	8	3.76	0.79	.715
Learn to train	7	3.13	0.66	.833
Train to train	10	3.47	0.73	.769
Train to Compete	6	1.79	0.38	.771
Compete to win	6	2.26	0.48	.734
Active for life	6	1.75	0.37	.809
Overall score	50	19.31	4.06	.763

Difficulty indices ranged from .328 to .686, and discrimination indices ranged from .367 to .768 (complete data shown above; results confirm that difficulty indices ranged between 0.328 and 0.686 and discrimination indices between 0.367 and 0.768, indicating acceptable levels.).

### Study variables

The independent variable was coaching certification level (Levels A, B, and C), representing formal coaching qualification categories within the national certification system.

### Data collection procedures

1. The validated and reliable test was used following expert review and pilot testing.
2. An official letter was issued by the Faculty of Physical Education to the Youth Leadership Preparation Center.
3. Another letter was sent from the Jordanian Olympic Committee to the Jordan Basketball Federation.
4. A pilot study was conducted on 14 basketball coaches. A pilot sample was considered sufficient for preliminary item analysis and reliability estimation in accordance with established psychometric guidelines.
5. Difficulty and discrimination analyses were performed.
6. The final electronic test was distributed through the Youth Leadership Preparation Center and the Jordan Basketball Federation.
7. To facilitate descriptive interpretation of results, the researchers adopted three benchmark categories commonly used in educational assessment contexts: (Table 3)

The electronic assessment required approximately 35–45 min to complete and was administered individually under standardized conditions. Participants completed the assessment remotely using an online platform distributed through the Jordan Basketball Federation and the Youth Leadership Preparation Center. Responses were automatically recorded and scored according to a predefined answer key established during instrument development.

**Table 3:** Knowledge level criteria.

Level	Description
Low	Less than 60%
Moderate	60%–80%
High	More than 80%

### Statistical analysis

Statistical analyses were performed using SPSS (IBM SPSS Statistics, Version 26). Independent samples *t*-tests were used to examine sex-based differences in LTAD knowledge, while one-way ANOVA was applied to evaluate differences across coaching certification levels. Effect sizes were calculated to determine the magnitude of differences. Cohen's *d* was used to estimate effect sizes for independent samples *t*-tests, while partial eta squared (*η*^2^*_p_*) was reported for ANOVA analyses. Normality and homogeneity of variance assumptions were assessed using Shapiro–Wilk and Levene's tests, respectively, and were satisfied for all analyses.

### Ethical approval

This study was conducted in accordance with the Declaration of Helsinki and approved by the institutional ethics committee of the authors' affiliated institution. Informed consent was obtained from all participants prior to data collection.

To enhance methodological rigor, the instrument was grounded in established LTAD theoretical frameworks and underwent expert validation and pilot testing prior to deployment. Data collection procedures were standardized, and statistical analyses were selected based on distributional assumptions to ensure robustness and reliability of findings.

## Results

### LTAD knowledge levels across developmental stages

Descriptive analysis revealed low biomechanics literacy across all LTAD developmental stages. The overall mean score was 24.52 out of 50 (SD = 9.44), representing 49.05% of the total possible score.

As shown in Table 4, the highest percentage score was observed in the Train to Train stage (52.86%), followed by the Train to Compete stage (51.19%) and the FUNdamentals stage (50.00%).

**Table 4:** Biomechanics literacy across LTAD stages among basketball coaches (*N* = 42).

No.	LTAD stage	No. of Items	Correct responses	*M* (SD)	% Score	Knowledge level
1	Active start	7	141	3.36 (1.79)	47.96	Low
2	FUNdamentals	8	168	4.00 (1.56)	50.00	Low
3	Learn to train	7	140	3.33 (1.97)	47.62	Low
4	Train to train	10	222	5.29 (2.28)	52.86	Low
5	Train to compete	6	129	3.07 (1.66)	51.19	Low
6	Compete to win	6	111	2.64 (1.71)	44.05	Low
7	Active for life	6	119	2.83 (1.74)	47.22	Low
	Total score	50	1,030	24.52 (9.44)	49.05	Low

SD, standard deviation; LTAD, long-term athlete development. Knowledge classification: <60% = low; 60%–80% = moderate; >80% = high.

Descriptive statistics [M (SD)] were calculated for all variables. Although standard deviations are reported, interpretation focused primarily on percentage classifications and mean scores, consistent with the study's objective of evaluating biomechanics literacy across LTAD stages.

Overall, percentage scores across all developmental stages remained below the predefined 60% benchmark for acceptable knowledge levels.

### Sex-based differences in LTAD knowledge

Welch independent-samples *t*-tests were conducted because of unequal group sizes between male and female coaches. As shown in Table 5, no statistically significant sex-based differences were observed across LTAD stages or total biomechanics literacy scores (*p* > .05). However, these findings should be interpreted cautiously due to the relatively small number of female participants.

**Table 5:** Sex-based differences in biomechanics literacy among basketball coaches.

LTAD stage	Male (*n* = 34) *M* (SD)	Female (*n* = 8) *M* (SD)	*t*(df)	*p*	Cohen's *d*
Active start	3.41 (1.69)	3.13 (2.30)	0.33 (8.87)	.747	0.16
FUNdamentals	4.15 (1.33)	3.38 (2.33)	0.90 (8.11)	.392	0.50
Learn to train	3.47 (2.05)	2.75 (1.58)	1.09 (13.18)	.295	0.36
Train to train	5.41 (2.18)	4.75 (2.76)	0.63 (9.15)	.543	0.29
Train to compete	3.12 (1.59)	2.88 (2.03)	0.32 (9.13)	.759	0.14
Compete to win	2.65 (1.77)	2.63 (1.51)	0.04 (12.04)	.972	0.01
Active for life	2.85 (1.65)	2.75 (2.19)	0.12 (8.97)	.903	0.06
Total score	25.06 (8.76)	22.25 (12.36)	0.61 (8.73)	.559	0.30

Welch independent-samples *t* tests were used because of unequal group sizes.

### Differences across coaching certification levels

As shown in Table 6, descriptive mean scores were relatively similar across certification groups. The one-way ANOVA results presented in Table 7 confirmed that these differences were not statistically significant.

**Table 6:** Descriptive biomechanics literacy scores by coaching certification level.

Certification level	*n*	Total score *M* (SD)
Level A	6	26.83 (12.06)
Level B	3	25.00 (11.36)
Level C	33	24.06 (9.07)
Total	42	24.52 (9.44)

**Table 7:** One-way ANOVA for biomechanics literacy across coaching certification level*s.*

Source	Sum of squares	df	Mean square	*F*	*p*	*η* ^2^ *_p_*
Between groups	39.76	2	19.88	0.21	.808	.011
Within groups	3,614.71	39	92.68			
Total	3,654.48	41				

*η*^2^_p_, partial eta squared.

### Domain-specific biomechanics literacy

Descriptive analysis of domain-specific biomechanics literacy revealed generally low scores across all assessed domains (Table 8). Coaches demonstrated relatively higher scores in load management (52.86%) and force production (50.00%). Lower scores were observed in neuromuscular development (44.05%), injury prevention (47.62%), and movement mechanics (47.96%). Overall, the findings suggest limited biomechanics literacy across both foundational and applied biomechanical domains.

**Table 8:** Domain-specific biomechanics literacy.

Domain	*M* (SD)	% Score
Movement mechanics	3.36 (1.79)	47.96
Force production	4.00 (1.56)	50.00
Injury prevention	3.33 (1.97)	47.62
Load management	5.29 (2.28)	52.86
Neuromuscular development	2.64 (1.71)	44.05
Kinetic chain integration	3.07 (1.66)	51.19

SD, standard deviation.

### Summary of findings

Overall, the findings demonstrated relatively low biomechanics literacy across LTAD developmental stages and biomechanical domains among basketball coaches. No statistically significant differences were observed across sex or coaching certification levels. Lower scores were particularly evident in advanced biomechanical domains associated with athlete development and training adaptation.

## Discussion

The present study examined biomechanics literacy among basketball coaches and its relevance to Long-Term Athlete Development (LTAD)-oriented coaching environments. The findings demonstrated relatively modest levels of biomechanics knowledge across all developmental stages, suggesting potential limitations in coaches' conceptual understanding of biomechanical principles relevant to athlete development. These findings align with pedagogical theories emphasizing the importance of domain-specific knowledge in shaping coaching and instructional practices (19, 20).

Although coaches demonstrated general familiarity with LTAD stages, lower performance across several biomechanical domains may indicate challenges in integrating scientific knowledge within coaching contexts. Previous research has similarly suggested that coaches may experience difficulty integrating theoretical knowledge into training environments (13, 21). However, it is important to note that the present study evaluated cognitive understanding of biomechanics principles rather than direct observation of coaching behavior or practical coaching effectiveness.

From an applied perspective, biomechanics literacy may contribute to coaches' understanding of movement mechanics, training design, and injury prevention strategies. The lower scores observed in the Train to Compete and Compete to Win stages may suggest reduced familiarity with advanced concepts such as load management, movement efficiency, and performance optimization within competitive environments.

Relatively lower scores were also observed in early developmental stages, including Active Start and FUNdamentals, which are important for developing movement competency and long-term athletic progression. Early identification and correction of movement-related limitations may represent an important developmental challenge within talent pathways and athlete support systems (22). Limited biomechanics understanding during these stages may reduce coaches' ability to optimally support coordination development, movement quality, and injury prevention within LTAD-oriented programs (9, 10). This interpretation is also consistent with the Youth Physical Development Model, which emphasizes age-appropriate physical development and long-term planning throughout athlete development pathways (23).

The absence of statistically significant differences across certification levels may indicate that current certification pathways do not consistently support the progressive development of applied biomechanics knowledge. However, factors such as course content, educational exposure, and time since certification were not directly assessed and therefore warrant further investigation. These findings are generally consistent with previous literature suggesting that formal coach education does not always result in measurable improvements in applied scientific understanding (16, 24). Effective coach development also requires attention to coaching pedagogy and the integration of scientific knowledge within practical coaching environments (25).

These findings may also reflect broader limitations within coaching systems where certification pathways emphasize administrative and managerial competencies more strongly than applied scientific monitoring and biomechanical analysis (26).

The domain-specific findings indicate relatively lower biomechanics literacy in areas associated with kinetic chain integration, neuromuscular development, and load management. These concepts are commonly linked to movement efficiency, training adaptation, and injury prevention within basketball environments (7, 8). The findings therefore highlight the potential value of strengthening applied biomechanics education within coach development pathways.

From a coaching science perspective, the findings highlight the importance of integrating biomechanics education within competency-based coach development frameworks and broader athlete development models (27, 28). Applied understanding of movement mechanics, load management, and injury prevention may support evidence-informed coaching practices and LTAD implementation.

In the Jordanian context, the findings may reflect broader challenges related to access to applied scientific resources and evidence-informed coach education opportunities. Similar patterns have been reported in other developing sport systems where coaching practice is frequently influenced by experiential learning and informal knowledge pathways (4).

Overall, this study contributes to the literature by providing empirical evidence regarding biomechanics literacy among basketball coaches within LTAD-oriented coaching environments. A key strength of the study lies in its structured LTAD-based assessment across multiple developmental stages, providing a domain-specific evaluation that remains relatively limited in coaching science research.

## Conclusion

This study identified relatively low levels of biomechanics literacy among basketball coaches, which may influence the implementation of Long-Term Athlete Development (LTAD) principles within coaching environments. No statistically significant differences were observed across coaching certification levels, suggesting limited differentiation in biomechanics literacy within the present sample. These findings highlight the importance of strengthening the integration of applied biomechanics within coach education programs through competency-based and evidence-informed learning approaches. Future research should further examine how biomechanics literacy relates to coaching behavior, athlete development, and practical coaching effectiveness.

### Limitations

This study is limited by its cross-sectional design, which restricts causal interpretation. The use of a cognitive assessment tool may not fully capture the practical application of biomechanics knowledge in real coaching environments. Additionally, the sample was limited to basketball coaches in Jordan, which may affect the generalizability of the findings. The relatively small sample size and gender imbalance should also be considered when interpreting the results. The relatively small subgroup sizes, particularly among female coaches, may have limited statistical power for subgroup comparisons.

Future research should incorporate observational and intervention-based approaches to better examine the application of biomechanical knowledge in coaching practice. It is important to note that the findings reflect associations rather than causal relationships due to the cross-sectional design.

## Data Availability

The data analyzed in this study is subject to the following licenses/restrictions: the dataset used and/or analyzed during the current study is not publicly available due to ethical and privacy considerations related to participant confidentiality. Data may be made available from the corresponding author upon reasonable request and with permission from the relevant institutional authority. Requests to access these datasets should be directed to hashemkilani@gmail.com.
